# Editorial: Therapeutic implications of circadian rhythms

**DOI:** 10.3389/fphar.2015.00175

**Published:** 2015-08-19

**Authors:** Guangrui Yang, Han Wang, Erquan Zhang

**Affiliations:** ^1^The Institute for Translational Medicine and Therapeutics, Perelman School of Medicine, University of PennsylvaniaPhiladelphia, PA, USA; ^2^Center for Circadian Clocks, Soochow UniversitySuzhou, China; ^3^National Institute of Biological SciencesBeijing, China

**Keywords:** circadian rhythm, circadian clocks, circadian disruption, therapeutic implications, metabolism, cardiovascular diseases, central nervous system, clinical relevance

Circadian rhythms are biological processes displaying endogenous and entrainable oscillations of about 24 h. In mammals the sleep/wake cycle, core body temperature fluctuation, and diurnal variation of blood pressure and heart rate are among the most well-known circadian rhythms. These rhythms are not just the consequence of activity/rest cycles, but are also controlled by molecular clocks, a biological network of fundamental value in the harmonization of physiological and biochemical processes with the external environment. Substantial evidence suggests that:

1. Dysregulation of the circadian system is a significant risk factor for many health problems such as metabolic disorders, cardiovascular diseases, impaired immune function, and accelerated aging. Restoring or strengthening the circadian rhythm may be therapeutic for these conditions.
2. The incidence and severity of many diseases, such as the onset of cardiovascular events, chronic obstructive pulmonary disease (COPD), inflammatory diseases, and mental disorders, are time-dependent.
3. The efficiency and side effects of many drugs has temporal variations.
4. Circadian rhythms can be modulated by some drugs.

Despite the large amount of experimental and epidemiological evidence, the importance of circadian rhythms has not been paid much attention in real clinical settings. The aim of this Research Topic in Frontiers is to highlight the therapeutic implications of circadian rhythms.

The mating behavior or close-proximity (CP) displays day/night variation in some insects including *Drosophila melanogaster*, which is controlled by molecular clocks and affected by food consumption. For example, CP rhythm is abolished in *per* or *tim*-null flies, and dampened under low-nutrient conditions. In the research article, *Inositols affect the mating circadian rhythm of Drosophila melanogaster* (Sakata et al., 2015), Sakata et al. found that CP rhythm significantly enhanced by feeding the flies with powdered ice plant, a little-known vegetable that may improve hyperglycemia in a streptozotocin-induced diabetic rat model. Among various components of ice plant, myo-inositol could increase the amplitude and shorten the period of CP rhythm. Real-time reporter assays showed that myo-inositol also shortened the period of the circadian reporter gene Per2-luc in the mouse cell line NIH3T3. Their data suggested that ice plant and myo-inositol may be beneficial to insect reproduction, while its potential role in mammals need to be carefully investigated.

There's obvious day/night variation in urinary voiding with much more during the day than at night in human. However, a large portion of human beings suffer excessive urination at night (nocturia), which dramatically decreases quality of life. Therefore, understanding the underlying mechanism has significant clinical relevance. Most studies on the circadian rhythm of micturition were focused on urine production by the kidneys. Although smooth muscle cells from mouse bladder express a functional and autonomous circadian clock at the molecular level, very few studies show circadian rhythms in the bladder function. In the research article, *Evaluation of mouse urinary bladder smooth muscle for diurnal differences in contractile properties* (White et al., 2014), White et al. measured spontaneous (phasic) and nerve-evoked contractions of mouse bladder tissue strips collected from multiple time points during 24 h and found phasic contraction, but not nerve-evoked contraction displayed diurnal rhythm.

Circadian rhythm has significant therapeutic implications in the central nervous system. It has long been known that circadian disruption by frequent shift work, jet lag, or exposure to artificial light is a risk factor for several neurodegenerative diseases, including Alzheimer's disease. Conversely, many neurodegenerative diseases result in circadian abnormalities. Besides, mice lacking clock genes, such as Bmal or Clock/Npas2, developed marked astrogliosis. In the mini-review, *Circadian clock disruption in neurodegenerative diseases: cause and effect?* (Musiek, 2015), Musiek reviewed recent studies implicating circadian rhythms and neurodegeneration and emphasized future research directions and potential therapeutic strategies for neurodegenerative diseases.

Cardiovascular disease (CVD) is a leading cause of death worldwide and new approaches in the management of CVD are clearly warranted. Since cardiovascular function and the onset of many CVDs display obvious diurnal variations, novel pharmacologic compounds that target the circadian mechanism may have potential clinical applications. Two review articles in current research topic were focused on the cardiovascular system and circadian rhythms from different views. In *Recent advances in circadian rhythms in cardiovascular system* (Chen and Yang, 2015), Chen and Yang summarized recent advances in the understanding of the relationship between circadian rhythm and cardiovascular physiology and diseases including blood pressure regulation and myocardial infarction. In *Therapeutic applications of circadian rhythms for the cardiovascular system* (Tsimakouridze et al., 2015), Tsimakouridze et al. mainly focused on circadian biomarkers, chronotherapy for CVDs and new drugs targeting circadian clocks.

Biological clock and metabolism are tightly intertwined. On one hand, the disturbance of circadian rhythms negatively affects metabolic homeostasis, and thus may promote the development of obesity and diabetes. On the other hand, high fat consumption alters circadian behavior in mice, while temporal restriction of food consumption limits mouse weight gain on a high fat diet via restoring the robustness of clock gene oscillation. In mini-review, *Circadian clocks, feeding time, and metabolic homeostasis* (Paschos, 2015), Paschos collected evidence about the association between circadian misalignment and metabolic homeostasis and discussed the role of feeding time in energy metabolism. In another review paper, *Rodent models to study the metabolic effects of shiftwork in humans* (Opperhuizen et al., 2015), Opperhuizen et al. provided a thorough view of animal models that are used to mimic human shiftwork. They divided published models in four categories, i.e., altered timing of food intake, activity, sleep, or light exposure and scored and compared their effects on metabolic parameters. They also discussed the drawback of animal studies and evaluated the translatability to human beings.

Mothers who experience breastfeeding problems in the early post-partum period are more likely to discontinue breastfeeding within 2 weeks. A major risk factor for shorter breastfeeding duration is delayed lactogenesis II (DLII). Based on the facts that circadian clocks coordinate hormonal and metabolic changes to support lactation in rodent studies, and disruption of the circadian system intervenes the initiation of lactation and negatively impacts milk production, Fu et al. (2015) hypothesized that DLII is related to disruption of the mother's circadian system. Authors reviewed literatures that support this hypothesis, and described interventions that may help to increase breastfeeding success.

The treatment of circadian disorders has drawn attention recently. However, the development of pertinent drugs has a high failure rate possibly due to the variations in chronotype. Therefore, similar to treatment of given cancers, personalized medicine might become a standard for drug development in the field of chronobiology. In *Personalized medicine for pathological circadian dysfunctions* (Skelton et al., 2015), Skelton et al. reviewed the current clinical trials of circadian drugs and the history of personalized medicine in oncology, and discussed how personalized medicine can be used in future clinical trials for circadian disorders.

As presented above, we recruited two research articles, six review articles, and a hypothesis and theory article that covered multiple aspects of *Therapeutic Implications of Circadian Rhythms*, from model organisms to human, from central nervous system to peripheral tissues, and from clinical study to drug development. Although much attention has been paid to this field in recent years, we are still far from our goal, especially the translation of basic science into clinical applications. Therefore, we encourage researchers to continue contributing to our understanding of the clinical relevance of circadian systems. Finally, we would like to thank all authors who contributed papers to our research topic. We would also like to thank all reviewers and editorial board for helping us to underscore the importance and organization of this Research Topic.

## Conflict of interest statement

The authors declare that the research was conducted in the absence of any commercial or financial relationships that could be construed as a potential conflict of interest.
